# The Role of Psychological Interventions in the Mental Health and Quality of Life of Older Adults: A Systematic Review with Meta-Analysis of Mindfulness, Cognitive Behavioral Therapy, and Reminiscence-Based Approaches

**DOI:** 10.3390/ejihpe16030034

**Published:** 2026-02-28

**Authors:** Paola Romera-Gasparico, María del Carmen Carcelén-Fraile, Javier Cano-Sánchez, Marcelina Sánchez-Alcalá, Juan Miguel Muñoz-Perete, Agustín Aibar-Almazán, Fidel Hita-Contreras, Yolanda Castellote-Caballero

**Affiliations:** 1 Department of Health Sciences, Faculty of Health Sciences, University of Jaén, 23071 Jaén, Spainaaibar@ujaen.es (A.A.-A.); mycastel@ujaen.es (Y.C.-C.); 2 Department of Educational Sciences, Faculty of Social Sciences, University of Atlántico Medio, 35017 Las Palmas de Gran Canaria, Spain

**Keywords:** psychological interventions, older adults, mindfulness, depression, anxiety, stress, loneliness, quality of life, meta-analysis

## Abstract

Psychological problems such as depression, anxiety, stress, loneliness, and reduced quality of life are prevalent in older adults, yet the effectiveness of psychological interventions remains heterogeneous. This systematic review with meta-analysis evaluated the impact of psychological and psychoeducational interventions on emotional symptoms and quality-of-life outcomes in adults aged 60 years and older. Following PRISMA 2020 guidelines, a comprehensive search was conducted in PubMed, Scopus, CINAHL, and Web of Science. Randomized controlled trials published in the last five years were included if they assessed interventions such as mindfulness, cognitive behavioral therapy, reminiscence therapy, or behavioral activation. Twenty-eight trials were included in the qualitative synthesis and twenty-two in the meta-analysis. Standardized mean differences (Hedges’ g) were pooled under fixed- and random-effects models. Heterogeneity, subgroup analyses, and publication bias were examined using Q, I^2^, Begg–Mazumdar, Egger, and Trim-and-Fill methods. The global meta-analysis showed a moderate and significant favorable effect of psychological interventions on emotional symptoms under the random-effects model (SMD = −0.623, 95% CI −0.888 to −0.359; *p* < 0.001), where negative values indicate reductions in symptom severity. Subgroup analyses revealed a moderate effect on depressive symptoms, which remained significant after adjustment for publication bias, and a large effect on perceived stress (SMD = 0.581; *p* < 0.001); for stress outcomes, positive SMDs indicate reductions in stress (i.e., improvement) after aligning scale directionality. Anxiety showed a significant effect only under the fixed-effects model, while loneliness showed a small but significant effect (SMD = −0.110; *p* = 0.018). Mindfulness-specific outcomes and quality of life did not show significant pooled effects. No substantial publication bias was detected. Psychological interventions significantly improve emotional well-being in older adults, particularly by reducing depression and stress. Effects on anxiety, loneliness, mindfulness, and quality of life are more variable, emphasizing the need for methodological consistency and longer follow-up in future studies.

## 1. Introduction

Population aging is one of the most significant demographic phenomena of the 21st century, driven by increased life expectancy and declining birth rates (Gianfredi et al., 2025). In the coming decades, the proportion of older adults is expected to surpass that of children, leading to profoundly aged societies and increased demands on health and social care systems (Khan et al., 2024). This demographic shift affects not only population structure but also care models, long-term care policies, and strategies to promote active and participatory aging (Buitendijk et al., 2025). Moreover, the accumulation of chronic conditions, multimorbidity, and functional limitations in later life increases pressure on healthcare systems and underscores the need for comprehensive approaches that address both physical and psychological well-being (Skou et al., 2022). This need has been particularly emphasized in older adults with chronic conditions and frailty, where non-pharmacological interventions have shown relevant effects on psychological outcomes (Tao et al., 2023). In this context, mental health in older adults emerges as a fundamental, though historically underserved, component of global public health (Reynolds et al., 2022).

As the proportion of older adults increases, so does the prevalence of various psychological problems that can profoundly affect the well-being of this population (Jalali et al., 2024). Among these, depression is one of the most frequent and often underdiagnosed disorders in old age, manifesting not only through persistent sadness but also through apathy, loss of interest, decreased activity levels, and somatic complaints (Devita et al., 2022). Its impact extends beyond mood, influencing the ability to perform daily activities and increasing the risk of functional disability (Goodarzi et al., 2024). Similarly, anxiety, although less recognized in older adults, is expressed through excessive worry, physical tension, somatic symptoms, and fear of losing autonomy, creating an emotional landscape that can interfere with psychological stability and daily life (Mohlman et al., 2012). These psychological conditions have been widely examined in recent systematic reviews and meta-analyses of psychological interventions for older adults, which report overall beneficial effects on mental health outcomes, while also highlighting substantial heterogeneity across intervention types and study designs (Iwano et al., 2022).

Another key component is stress, which in old age often stems from factors accumulated throughout life and significant transitions such as retirement, changes in social roles, interpersonal losses, or the onset of chronic illnesses (Majnarić et al., 2021). Chronic stress not only affects emotional well-being but also exacerbates underlying medical conditions, increases physical vulnerability, and contributes to cognitive decline (Mariotti, 2015). Associated with this, perceived loneliness and social isolation have been identified as particularly prevalent experiences at this stage and can increase the risk of depression, anxiety, and impaired sleep quality (Brandt et al., 2022). Loneliness, understood as the discrepancy between desired and available social relationships, represents a critical risk factor, closely linked to a decline in psychological well-being (Puyané et al., 2025). In contrast, positive variables such as mindfulness have gained increasing relevance as they are studied as an internal resource that can promote emotional self-regulation, stress reduction, and adaptation to the changes associated with aging (Javadzade et al., 2024). Evidence from previous reviews suggests that positive psychological interventions contribute significantly to subjective well-being and emotional health in older adults (Sutipan et al., 2017). The ability to maintain conscious and non-judgmental attention to the present experience has been linked to fewer emotional symptoms, greater resilience, and better indicators of subjective well-being in older adults (Alkan et al., 2025). All of these factors have a direct impact on quality of life, a multifactorial construct encompassing physical, emotional, social, and functional dimensions (Nouri et al., 2021). In old age, quality of life is affected by the presence of psychological symptoms, as well as by perceived control, opportunities for social participation, sense of purpose, and life satisfaction (Lopez et al., 2024). When depression, anxiety, stress, or loneliness intensify, quality of life tends to deteriorate significantly, affecting autonomy, motivation, social interaction, and overall health (Holt-Lunstad, 2024). Importantly, although depression, anxiety, stress, loneliness, and quality of life are often examined as distinct constructs, they are conceptually and empirically interrelated, particularly in later life. Stressful life events and chronic stress can increase vulnerability to anxiety and depressive symptoms, while persistent emotional distress may, in turn, undermine perceived quality of life and functional well-being. Conversely, lower quality of life and reduced social participation can exacerbate emotional symptoms, creating reciprocal and mutually reinforcing processes. From a clinical and research perspective, examining these outcomes both independently and jointly allows for a more nuanced understanding of mental health in older adults, acknowledging their overlap while preserving their conceptual specificity. The interaction between these dimensions and physical health is particularly close in old age, given that emotional problems can exacerbate chronic illnesses, hinder adherence to healthy habits, and accelerate functional decline. In this context, it becomes essential to examine the available therapeutic responses to address these emerging needs (Basu et al., 2023).

Although pharmacological treatments have traditionally been one of the main clinical strategies for addressing mental health in older adults, their exclusive use presents several limitations that have been widely noted in the scientific literature, such as increased physiological susceptibility and polypharmacy (Ngcobo, 2025). Many older adults take multiple medications to treat chronic conditions, which increases the risk of interactions, adverse reactions, and adherence problems (Varghese et al., 2025). Therefore, in recent years, there has been a notable increase in interest in psychological interventions aimed at promoting active, independent, and healthy aging (Davodi et al., 2023). Importantly, different types of psychological interventions may influence mental health outcomes in older adults through partially distinct mechanisms. Mindfulness-based approaches primarily target attentional control, emotional awareness, and acceptance, which may help reduce stress reactivity and rumination. Cognitive behavioral therapy focuses on identifying and modifying maladaptive thought patterns and behaviors, thereby directly addressing depressive and anxiety symptoms through cognitive restructuring and behavioral activation. In contrast, reminiscence-based interventions emphasize autobiographical memory, meaning-making, and life review processes, which may be particularly relevant for emotional integration, self-esteem, and well-being in later life. These conceptual differences provide a theoretical rationale for examining intervention-specific effects and justify the use of subgroup analyses to explore potential variability in outcomes across intervention types. In particular, low-intensity psychological interventions have been highlighted as feasible and acceptable approaches with positive effects on well-being in later life (Cremers et al., 2022). These approaches focus not only on reducing psychological symptoms but also on strengthening internal resources, improving coping skills, fostering social integration, and enhancing a sense of purpose, self-efficacy, and life satisfaction (Ayoubi-Mahani et al., 2023). They also seek to support the maintenance of cognitive functions, encourage participation in meaningful activities, and offer strategies that help older adults adapt more effectively to the changes inherent in this stage of life. Taken together, these interventions represent an essential complement to traditional care models, responding more comprehensively to the emotional, social, and cognitive needs of the elderly population (Oberlin et al., 2022).

Therefore, the objective of this systematic review with meta-analysis was to evaluate the effectiveness of psychological interventions in older adults on key mental health outcomes, specifically depression, anxiety, perceived stress, loneliness, and mental health-related quality of life. In addition, subgroup analyses were conducted to explore whether intervention effects varied across different types of psychological approaches (e.g., mindfulness-based, cognitive behavioral, and reminiscence-based interventions) and outcome domains, based on theoretical differences in their underlying mechanisms of action.

## 2. Materials and Methods

This review was carried out in accordance with the 2020 PRISMA statement guidelines (Page et al., 2021, see Table S1 for the PRISMA checklist) and was based on a protocol previously registered in PROSPERO (CRD420251236372). Moreover, its methodological approach followed the recommendations provided in the Cochrane Handbook for Systematic Reviews of Interventions (Higgins et al., 2024).

### 2.1. Sources of Information

An extensive literature search was conducted from October to November 2025 using the PubMed, Scopus, CINAHL, and Web of Science (WOS) databases.

### 2.2. Search Strategy

Multiple search terms were utilized in the following search string: (“psychological intervention” OR “psychotherapy” OR “cognitive behavior therapy” OR “mindfulness” OR “reminiscence therapy”) AND (“mental health” OR “depression” OR “anxiety” OR “stress” OR “quality of life”) AND (“older adults” OR “elderly” OR “older people” OR “geriatric”).

### 2.3. Inclusion Criteria

The articles selected for this review were required to meet the following criteria: (i) the studies had to be randomized controlled trials (RCTs); (ii) the intervention must involve a psychological approach, such as cognitive behavioral therapy, mindfulness, psychotherapy, or reminiscence therapy; (iii) participants had to be older adults aged 60 years or above; and (iv) the studies must assess mental health outcomes, specifically depression, anxiety, stress, or mental health–related quality of life. Additionally, (v) only studies published within the last five years were considered. This temporal restriction was applied to ensure that the review reflects the most current evidence in a rapidly evolving field, characterized by ongoing methodological refinements, the development of novel intervention formats (e.g., low-intensity and digitally delivered therapies), and changes in clinical practice. Moreover, limiting inclusion to recent studies helps reduce redundancy with previous systematic reviews and meta-analyses while providing an updated synthesis of contemporary psychological interventions for older adults.

### 2.4. Exclusion Criteria

Articles were excluded if they met any of the following criteria: (i) studies lacking a control or comparison group; (ii) studies that did not evaluate mental health outcomes relevant to this review; (iii) interventions that were not psychological in nature, such as pharmacological, physical, or nutritional interventions; (iv) studies involving mixed-age samples in which data for older adults were not reported separately; studies in which participants had a primary diagnosis of moderate-to-severe neurocognitive disorder/dementia (as defined and reported by the trial authors, e.g., DSM/ICD or clinical diagnosis), or where the intervention primarily targeted dementia-related cognitive outcomes; when reported, cognitive screening thresholds (e.g., MMSE/MoCA cutoffs) were used to inform eligibility decisions; and (vi) studies involving participants with significant physical pathologies when these conditions were central to the study or could substantially influence mental health outcomes.

### 2.5. Study Selection Process

The study selection process began by removing duplicate records and articles without accessible abstracts. Titles and abstracts were then screened to exclude studies that did not meet the predefined eligibility criteria. The remaining articles were examined in full to determine their suitability for inclusion in the meta-analysis. To ensure impartiality and minimize bias, two authors (J.C-S. and M.d.C.C.-F.) independently carried out the screening. Any disagreements regarding study eligibility were resolved through consultation with a third author (P.R.-G.), who facilitated consensus. This thorough procedure ensured that all included studies were relevant and met the established criteria.

### 2.6. Data Extraction

The primary variables in this review were mental health outcomes in older adults, specifically depression, anxiety, stress, loneliness, mindfulness attention awareness, and mental health–related quality of life. The data extraction process involved collecting information on authors, year of publication, study setting, participant characteristics (sample size, age, and group allocation), study design, details of the psychological interventions, outcome measures and instruments used, timing of evaluations, and the key findings reported in each study.

### 2.7. Assessment of Methodological Quality

The methodological quality of the studies was assessed using the PEDro scale (Cashin & McAuley, 2020), an 11-item checklist. The highest achievable score is 10 points, as the first item (“eligibility criteria”) is excluded from the final rating. Each criterion is marked as “Yes” (1 point) or “No” (0 points). Quality is categorized as follows: scores from 0 to 3 denote “Poor” quality, scores of 4 to 5 indicate “Fair” quality, scores between 6 and 8 reflect “Good” quality, and scores above 9 correspond to “Excellent” quality. Additionally, the Cochrane RoB-2 tool was employed to assess the risk of bias in the included studies. This instrument is tailored to evaluate sources of bias in randomized research, particularly randomized clinical trials. It reviews five main domains and assigns each study a risk level of low, high, or unclear (Sterne et al., 2019).

### 2.8. Analytic Decisions for Meta-Analysis

The results are presented through a forest plot, which includes the first author, total sample size. Standardized mean differences (SMDs) were calculated using Hedges’ g, which applies a correction for small sample bias and the pooled effect size with its 95% confidence interval and corresponding *p*-value. Given the expected clinical and methodological heterogeneity across psychological intervention studies, random-effects models were specified a priori as the primary analytic approach. Fixed-effects models were additionally computed for comparative purposes. Statistical heterogeneity was formally assessed using the Cochrane Q test and the I^2^ statistic (Higgins et al., 2023). For subgroup or stratified analyses, studies were grouped according to the type of intervention, and separate meta-analyses were carried out for each group. This approach allowed for a more detailed examination of variability and effect sizes across subgroups, improving the clarity of the findings.

Effect sizes were coded to ensure that values consistently reflected improvement in the target outcome. Accordingly, negative standardized mean differences (Hedges’ g) indicate symptom reduction favoring the intervention group for outcomes such as depression, anxiety, and loneliness. For stress outcomes, several instruments (e.g., Perceived Stress Scale) are scored such that higher values indicate greater perceived stress. Therefore, stress outcomes were coded so that positive standardized mean differences represent reductions in stress levels favoring the intervention group after aligning scale directionality. This recoding was applied only when necessary and followed standard meta-analytic conventions to facilitate comparability across outcomes. Finally, a funnel plot was generated to explore the presence of potential publication bias.

## 3. Results

**Figure 1:**
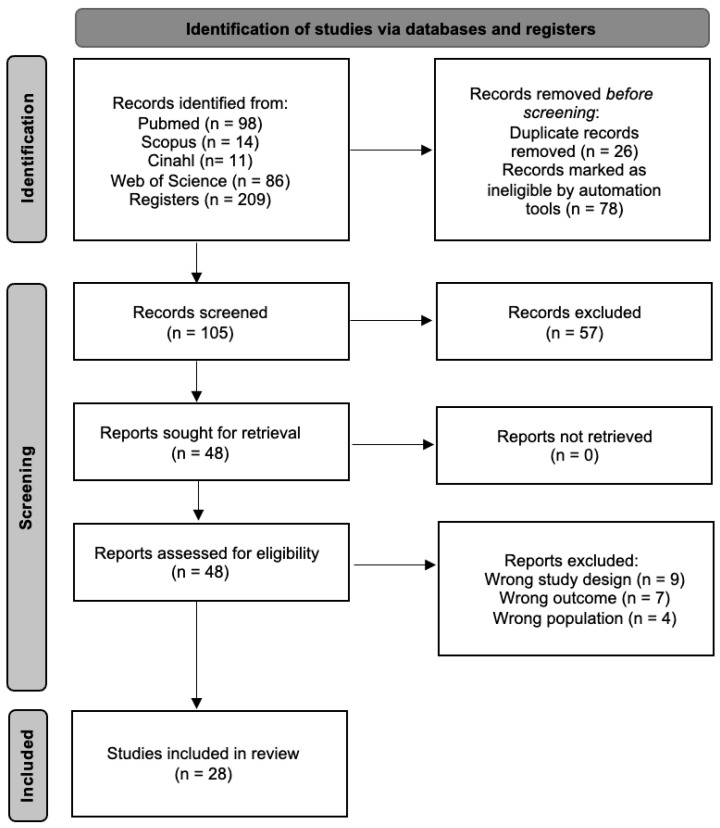
Study selection process flow chart.

An initial search across multiple databases identified 209 articles. The search was then refined within the same databases by targeting specific document types (articles and randomized clinical trials) and filtering for keywords in titles and abstracts, while also removing duplicates. This process resulted in 105 unique articles. These articles were then screened based on their titles and abstracts, narrowing the selection to 48 articles for full-text assessment. Following full-text evaluation, 28 articles met the inclusion criteria and were included in the meta-analysis, while the remaining 20 full-text articles were excluded for not fulfilling the eligibility criteria. The selection process is detailed further in Figure 1.

### 3.1. Methodological Quality

The methodological quality of the studies included was assessed using the PEDro scale, with scores obtained from the PEDro website. 13 articles were rated as “Fair”, one as “Poor”, while the remaining studies were classified as “Good”. To maintain objectivity and consistency in the evaluation, two independent reviewers scored the studies based on the PEDro scale. In cases of disagreement, a conflict resolution procedure was followed. The reviewers discussed the differences, and if they could not reach a consensus, a third reviewer was involved. This approach ensured that the study grading was as accurate and reliable as possible. A comprehensive assessment of the methodological quality can be found in Table 1.

The risk of bias was evaluated using the Cochrane RoB-2 tool, classifying each study as having a low risk, some concerns, or a high risk of bias. Of the 28 included studies, 5 were rated as having a low risk of bias, 17 were judged as presenting some concerns, and the remaining 6 were classified as having a high or mixed (some concerns/high) overall risk of bias. A detailed domain-level assessment for each study is presented in Table 2.

### 3.2. Characteristics of the Studies

All studies included in this systematic review and meta-analysis were randomized controlled trials conducted in Israel (Aisenberg-Shafran & Shturm, 2022; Shapira et al., 2021), the United States (Alexopoulos et al., 2021; Dutcher et al., 2025; Lindsay et al., 2022; Lwi et al., 2023; Van Orden et al., 2021), Germany (Dafsari et al., 2023), Japan (Hanaoka et al., 2025; Komase et al., 2025), Iran (Javadzade et al., 2024; Javadzade et al., 2025), China (Jiang et al., 2025; Kwok et al., 2024; Lee et al., 2022; S. Li et al., 2022; Pan et al., 2024; Shapira et al., 2021; Wu et al., 2025), Turkey (Kabataş Yıldız & Orak, 2023) Canada (Landreville et al., 2025; Sadowski et al., 2025), the Netherlands (Lely et al., 2022), Brazil (Mapurunga et al., 2025), Sweden (Nordgren et al., 2024), Thailand (Phoobangkerdphol et al., 2022) and Spain (Rodríguez-Romero et al., 2021). A total of 4704 participants took part in these studies, with 1870 in the control group and 2834 in the intervention group, which focused on physical activity. There was a higher representation of women among the total participants included in the analyzed studies. The average age of participants was 72.94 years (Table 3).

### 3.3. Study Results

Of the 28 articles included in this systematic review, all were considered in the meta-analysis. The main objective of this review is to assess anxiety, depression, loneliness, mindfulness, stress, and quality of life in older adults. Anxiety was examined in 5 articles (Dafsari et al., 2023; Landreville et al., 2025; Phoobangkerdphol et al., 2022; Shorey et al., 2021; Van Orden et al., 2021) using the Geriatric Anxiety Inventory (GAI) and the Generalized Anxiety Disorder 7-item (GAD-7). Depression was assessed in 19 articles (Aisenberg-Shafran & Shturm, 2022; Alexopoulos et al., 2021; Hanaoka et al., 2025; Javadzade et al., 2024; Javadzade et al., 2025; Jiang et al., 2025; Kabataş Yıldız & Orak, 2023; Komase et al., 2025; Kwok et al., 2024; Landreville et al., 2025; Lee et al., 2022; Lwi et al., 2023; Nordgren et al., 2024; Rodríguez-Romero et al., 2021; Shapira et al., 2021; Shorey et al., 2021; Van Orden et al., 2021; Wu et al., 2025) using the Geriatric Depression Scale (GDS), the Patient Health Questionnaire (PHQ-9), and the 24-item Hamilton Depression Rating Scale (HAM-D). Loneliness was analyzed in 7 studies (Dutcher et al., 2025; Hanaoka et al., 2025; Jiang et al., 2025; Kwok et al., 2024; S. Li et al., 2022; Lindsay et al., 2022; Rodríguez-Romero et al., 2021) using the UCLA Loneliness Scale. Mindfulness was evaluated in 3 trials (Lwi et al., 2023; Mapurunga et al., 2025; Shorey et al., 2021) using the Mindfulness Attention Awareness Scale (MAAS). Stress levels were studied in 4 articles (Jiang et al., 2025; Kabataş Yıldız & Orak, 2023; S. Li et al., 2022; Sadowski et al., 2025) using the Perceived Stress Scale (PSS). Finally, quality of life was assessed in 6 articles (Dafsari et al., 2023; Lely et al., 2022; Mapurunga et al., 2025; Pan et al., 2024; Shorey et al., 2021) using both the World Health Organization Quality of Life-BREF (WHOQOL-BREF) and WHOQOL-OLD.

In relation to anxiety, a statistically significant effect was found only in the study by Dafsari et al. (Dafsari et al., 2023), which reported a statistically significant reduction in anxiety (with a *p* < 0.05 value). In contrast, the remaining trials did not reach statistical significance (*p* > 0.05), suggesting that the interventions assessed in those studies did not substantially influence anxiety outcomes.

Regarding depressive symptoms, significant results were identified in several of the studies included in the meta-analysis, specifically those conducted by Javadzade et al. (Javadzade et al., 2024, 2025), Jiang et al. (2025), Kabataş Yıldız and Orak (2023), Kwok et al. (2024), Landreville et al. (2025), Nordgren et al. (2024), and Wu et al. (2025), all of which reported statistically significant reductions in depression levels (*p* < 0.05). By contrast, the remaining articles failed to achieve statistical significance (*p* > 0.05), indicating that the interventions did not exert a measurable impact on depressive symptomatology.

With respect to loneliness, significant effects were observed in the studies by Hanaoka et al. (2025), Jiang et al. (2025), Kwok et al. (2024), S. Li et al. (2022), and Rodríguez-Romero et al. (2021), all demonstrating statistically significant reductions in loneliness (*p* < 0.05). Conversely, the findings from Dutcher et al. (2025) and Lindsay et al. (2022) did not show significant changes, reflecting a limited influence of the interventions on perceived loneliness.

The results of the individual studies assessing mindfulness showed mixed findings. Lwi et al. (2023) and Mapurunga et al. (2025) reported non-significant effects (*p* > 0.05), indicating that the mindfulness interventions did not produce measurable changes in the outcomes evaluated. In contrast, Shih et al. (2021) found a statistically significant reduction (*p* = 0.020), suggesting that mindfulness may have had a meaningful impact in this particular trial.

In the case of stress, the evidence from the individual studies was consistently favorable. Jiang et al. (2025), Kabataş Yıldız and Orak (2023), S. Li et al. (2022), and Sadowski et al. (2025) all reported statistically significant reductions in stress levels (*p* < 0.05), indicating that the interventions applied in each trial produced meaningful improvements in this outcome.

Pertaining to quality of life, statistically significant results were identified in several of the studies included in the meta-analysis, specifically those conducted by Mapurunga et al. (2025), Pan et al. (2024), and Phoobangkerdphol et al. (2022), all of which reported a statistically significant impact of the intervention (*p* < 0.05). In contrast, the remaining articles, namely those by Dafsari et al. (2023), Lely et al. (2022), and Shorey et al. (2021), failed to achieve statistical significance (*p* > 0.05), suggesting that the interventions in these studies did not exert a measurable impact on quality of life.

### 3.4. Meta-Analysis

**Figure 2:**
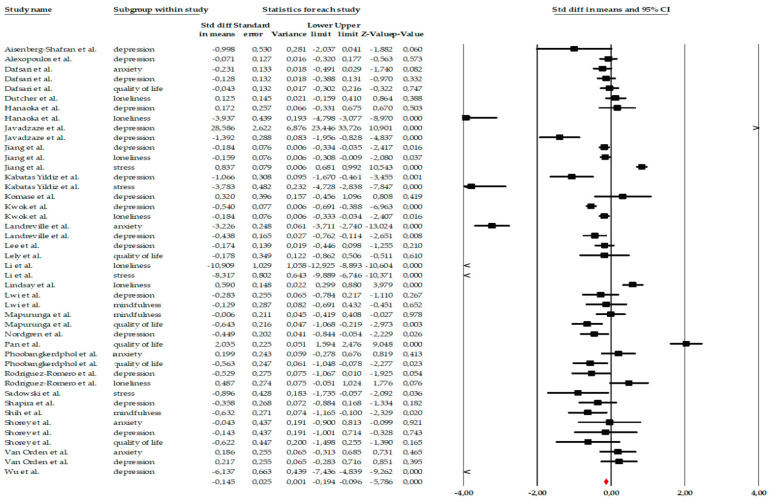
Forest plot of the global meta-analysis on the effects of psychological Interventions in the mental health and quality of life in older adults, showing pooled effect sizes under the random-effects model (Hedges’ g), with 95% confidence intervals. Black squares represent the standardized mean difference (SMD) for each individual study, with the size of the square proportional to the study weight. Horizontal lines indicate the 95% confidence intervals (CI). The red diamond represents the pooled effect estimate, and its width corresponds to the 95% CI. The vertical line at zero indicates no effect.

28 studies were included in the meta-analysis, evaluating the effect of psychological and psychoeducational interventions (such as mindfulness, CBT, reminiscence, or behavioral activation) on emotional variables in older adults. The analysis was performed using the standardized mean difference (SMD) as a measure of effect size. Under a fixed-effects model, a small but significant effect favoring the interventions was observed (SMD = −0.145, 95% CI −0.194 to −0.096; *p* < 0.001). However, given the extremely high heterogeneity among the studies (Q = 1072.93; *p* < 0.001; I^2^ = 95.99%), the most appropriate interpretation corresponds to the random-effects model. Under the random-effects model, the interventions showed a moderate and significant effect, with an effect size of SMD = −0.623, 95% CI = −0.888 to −0.359, *p* < 0.001. This pooled estimate should not be interpreted as a single uniform effect size, but rather as an indicator of the overall direction and consistency of effects across a highly diverse body of interventions, outcomes, and populations. The high heterogeneity (I^2^ ≈ 96%) suggests substantial variability among the studies, likely due to differences in intervention types, duration, intensity, population characteristics, and measured outcomes. The tau-square value (τ^2^ = 0.690) confirms the presence of real variability among effect sizes beyond sampling error (Figure 2). Accordingly, the global meta-analytic estimate is complemented by subgroup analyses and narrative synthesis, which together allow for a more nuanced interpretation of the findings. Overall, the analysis demonstrates that psychological and psychoeducational interventions have a positive and significant impact on the emotional well-being of older adults, although with notable heterogeneity among the included studies.

#### 3.4.1. Subgroup Analysis

##### Anxiety

**Figure 3:**
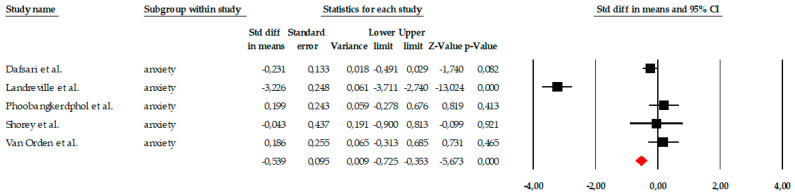
Forest plot of the meta-analysis on the effects of psychological interventions on anxiety in older adults, showing pooled effect sizes under the random-effects model (Hedges’ g), with 95% confidence intervals. Black squares represent the standardized mean difference (SMD) for each individual study, with the size of the square proportional to the study weight. Horizontal lines indicate the 95% confidence intervals (CI). The red diamond represents the pooled effect estimate, and its width corresponds to the 95% CI. The vertical line at zero indicates no effect.

The meta-analysis included five studies that evaluated the impact of different psychological interventions on anxiety levels in older adults. Overall, individual effect sizes ranged from small to very large reductions, with standard error of measurement (SEM) values from −0.04 to −3.13, consistently favoring the interventions over the control group. When the entire set of studies was analyzed using a fixed-effects model, the intervention showed a moderate and statistically significant effect on anxiety (SEM = −0.539, standard error = 0.095), with a 95% confidence interval ranging from −0.725 to −0.353. The associated Z-value was −5.673 (*p* < 0.001), indicating a clear reduction in anxiety symptoms in the treatment groups. However, this effect should be interpreted with caution due to the extremely high heterogeneity among the studies (Q = 141.67; *p* < 0.001; I^2^ = 97.18%), which indicates substantial clinical and methodological differences among the included research. For this reason, analysis using a random-effects model was considered more appropriate. In this model, although the effect size continued to point toward a benefit from the intervention, it no longer reached statistical significance. The overall effect size was SMD = −0.629, with a wide confidence interval (95% CI = −1.848 to 0.590) and a Z-value of −1.011 (*p* = 0.312). The magnitude of heterogeneity remained high (τ^2^ = 1.857), suggesting that the differences were not solely due to chance, but rather to real variations between studies, such as the type of intervention (mindfulness, CBT, reminiscence), its intensity, or the target population (Figure 3).

**Figure 4:**
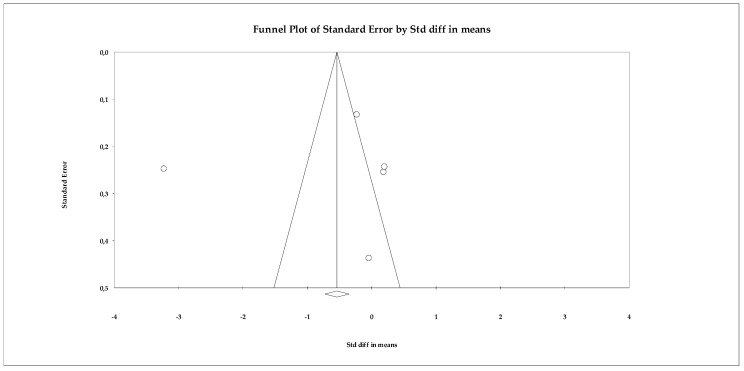
Funnel plot for the assessment of publication bias in the anxiety meta-analysis, based on effect sizes (Hedges’ g) from the random-effects model.

Regarding publication bias, the funnel plot showed some skewness, but statistical tests did not detect significant bias. The Begg–Mazumdar test yielded a tau of −0.60 (uncorrected) with *p* = 0.141, indicating no robust evidence of bias. The Trim and Fill analysis estimated only one missing study and slightly adjusted the effect size (adjusted SMD = −0.672, 95% CI −0.846 to −0.498), without changing the direction of the result (Figure 4).

##### Depression

**Figure 5:**
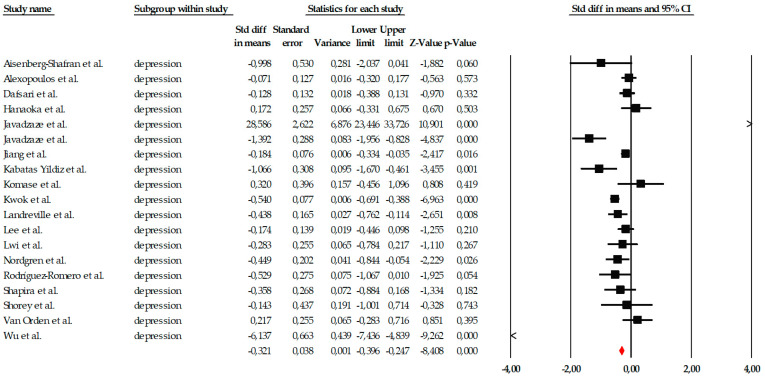
Forest plot of the global meta-analysis on the effects of psychological interventions on depressive symptoms, based on the random-effects model. Black squares represent the standardized mean difference (SMD) for each individual study, with the size of the square proportional to the study weight. Horizontal lines indicate the 95% confidence intervals (CI). The red diamond represents the pooled effect estimate, and its width corresponds to the 95% CI. The vertical line at zero indicates no effect.

The analyses of the depression outcome included nineteen studies evaluating the effects of psychological interventions on depressive symptoms in older adults. Individual effect sizes ranged from small to very large reductions, reflecting substantial variability across interventions and study populations. Given the extremely high heterogeneity observed (Q ≈ 250.6; *p* < 0.001; I^2^ ≈ 93%), the random-effects model was considered the primary and most appropriate analytical approach. Under this model, psychological interventions showed a moderate and statistically significant reduction in depressive symptoms, with a pooled effect size of SMD = −0.56 (95% CI −0.74 to −0.42; *p* < 0.001) (Figure 5). For completeness, a fixed-effects model was also calculated, yielding a smaller but statistically significant effect (SMD = −0.321, 95% CI −0.396 to −0.247; *p* < 0.001); however, this estimate should be interpreted with caution given the violation of the homogeneity assumption.

**Figure 6:**
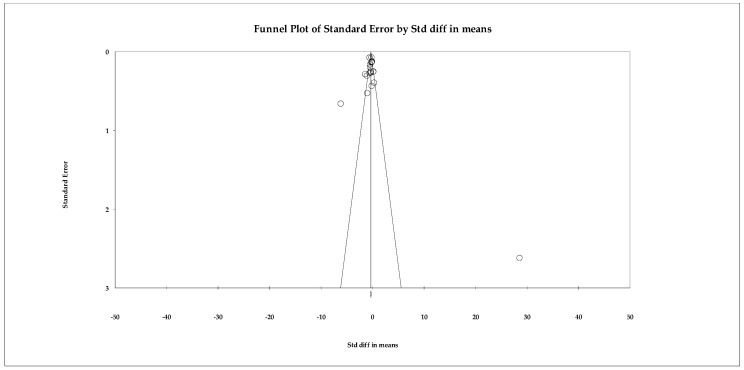
Funnel plot for the meta-analysis of depressive symptoms, used to assess publication bias.

Publication bias analyses suggested robust findings. Although the funnel plot showed some visual asymmetry, neither the Begg–Mazumdar test (τ = −0.21; *p* = 0.207) nor Egger’s regression (intercept = 0.38; *p* = 0.810) indicated significant bias. The Trim-and-Fill method identified one potentially missing study, but the adjusted random-effects estimate remained within the same moderate range and did not alter the direction or clinical relevance of the effect (Figure 6). The fail-safe N indicated that 185 null studies would be required to negate the observed effect.

##### Loneliness

**Figure 7:**
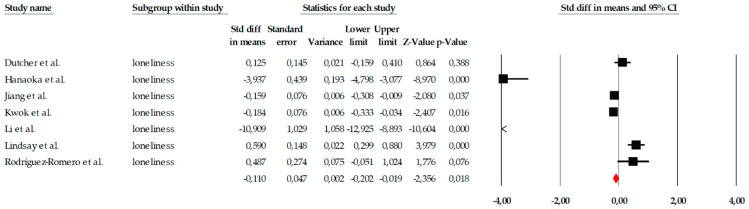
Forest plot of the meta-analysis on the effects of psychological interventions on loneliness, based on the random-effects model. Black squares represent the standardized mean difference (SMD) for each individual study, with the size of the square proportional to the study weight. Horizontal lines indicate the 95% confidence intervals (CI). The red diamond represents the pooled effect estimate, and its width corresponds to the 95% CI. The vertical line at zero indicates no effect.

The meta-analysis on the variable of loneliness included a total of seven studies, which evaluated the impact of different psychological interventions, primarily mindfulness programs, behavioral activation, reminiscence therapy, and group therapies, aimed at reducing the perception of loneliness in older adults. Individual effect sizes showed considerable variability, ranging from small effects (e.g., SMD = −0.184) to very large effects, such as that observed in Hanaoka et al. (2025) (SMD = −3.937), demonstrating relevant differences in the intensity, duration, and therapeutic approach of the interventions examined (Figure 7). When the studies were combined using a fixed-effects model, the overall effect size was small but statistically significant (SMD = −0.110; 95% CI: −0.202 to −0.019; *p* = 0.018), indicating that, on average, the interventions had a positive effect on reducing loneliness. This estimate, along with the notable dispersion in individual effect sizes, can be observed in the corresponding forest plot (Figure 7). However, the heterogeneity analysis revealed a Q value of 217.21, with *p* < 0.001 and I^2^ = 97.23%, indicating extremely high heterogeneity among the studies. Due to this degree of inconsistency between trials, the overall effect was also estimated using a random-effects model, yielding a larger effect size (SMD = −1.083; 95% CI: −1.738 to −0.429; *p* = 0.001). Although this value suggests that the interventions could have a relevant impact in some specific contexts, the wide confidence interval also reflects the substantial variability between studies, justifying a cautious interpretation of the effect.

**Figure 8:**
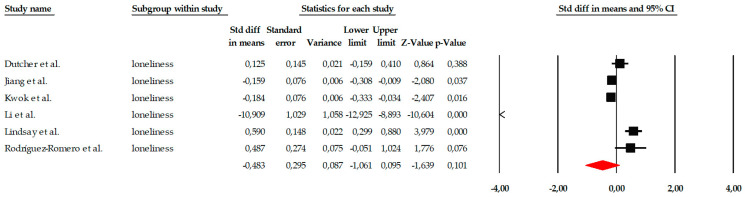
Sensitivity analysis forest plot for loneliness excluding Hanaoka et al. Black squares represent the standardized mean difference (SMD) for each individual study, with the size of the square proportional to the study weight. Horizontal lines indicate the 95% confidence intervals (CI). The red diamond represents the pooled effect estimate, and its width corresponds to the 95% CI. The vertical line at zero indicates no effect.

Given the presence of an extreme effect size in Hanaoka et al. (2025) (SMD = −3.937), a sensitivity analysis was conducted excluding this study to assess the robustness of the pooled estimate. After exclusion, the pooled random-effects effect size decreased from SMD = −1.083 (95% CI −1.738 to −0.429; *p* = 0.001) to SMD = −0.483 (95% CI −1.061 to 0.095; *p* = 0.101). The result no longer reached statistical significance. Heterogeneity remained high (I^2^ = 96.44%), indicating that substantial between-study variability persisted even after removal of the outlier. These findings suggest that the overall estimate for loneliness is sensitive to the inclusion of extreme effect sizes and should therefore be interpreted with caution (Figure 8).

**Figure 9:**
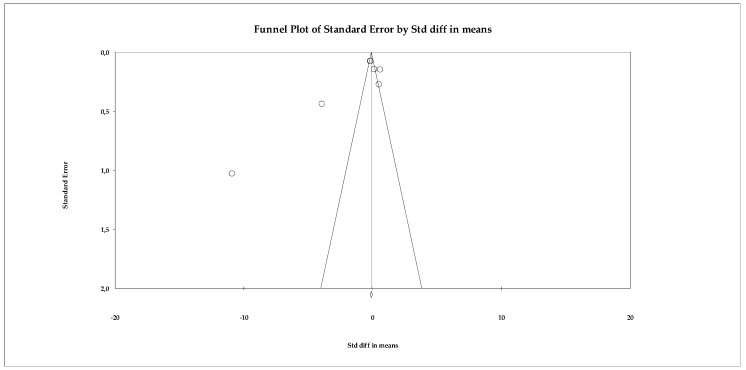
Funnel plot for the meta-analysis of loneliness, used to assess publication bias.

Publication bias analyses were conducted using the primary dataset including all eligible studies. The publication bias analysis showed that, although the funnel plot exhibited a slight visual asymmetry, this pattern was not confirmed by statistical tests, which failed to reach significance. As shown in Figure 9, most studies are distributed relatively symmetrically around the estimated effect, with no clear evidence of a systematic absence of small studies. The Begg–Mazumdar test yielded a tau of −0.238 (*p* = 0.452), while the Egger regression showed an intercept of −5.562 (two-tailed *p* = 0.190), indicating no detectable bias. The fail-safe N calculation showed that 73 studies with a null effect would be needed to invalidate the statistical significance of the original pooled estimate (*p* < 0.05), suggesting moderate robustness of the finding. However, sensitivity analyses indicated that the pooled estimate for loneliness may be influenced by extreme effect sizes and should therefore be interpreted cautiously. Furthermore, the Trim and Fill method did not identify any missing studies, so both the observed and adjusted effects coincide (SMD = −0.110 in both cases).

##### Mindfulness

**Figure 10:**
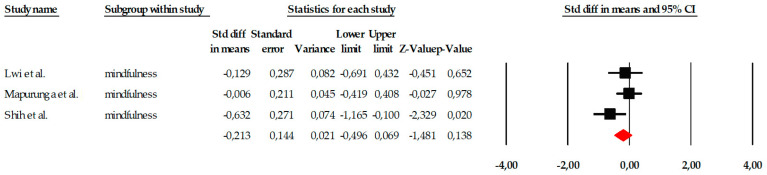
Forest plot of the meta-analysis on the effects of psychological interventions on mindfulness, based on the random-effects model. Black squares represent the standardized mean difference (SMD) for each individual study, with the size of the square proportional to the study weight. Horizontal lines indicate the 95% confidence intervals (CI). The red diamond represents the pooled effect estimate, and its width corresponds to the 95% CI. The vertical line at zero indicates no effect.

The subgroup of mindfulness consisted of three studies, which represents the minimum number required to conduct a quantitative synthesis and is therefore methodologically acceptable. However, the very limited number of included trials substantially restricts the strength and reliability of the conclusions that can be drawn from this subgroup analysis. Although the variability in individual effect sizes allowed for pooled estimation under a fixed-effects model, the small evidence base increases statistical uncertainty and limits generalizability. The included studies showed heterogeneous results: while two reported very small or virtually no effects (e.g., SMD = −0.006 in Mapurunga et al., 2025), the study by Shih et al. (2021) reported a moderate effect in a favorable direction (SMD = −0.632; *p* = 0.020). This distribution can be seen graphically in the corresponding forest plot (Figure 9). When the results were combined under a fixed-effects model, the overall effect size indicated a small and non-significant reduction in the assessed symptoms (SMD = −0.213; 95% CI: −0.496 to 0.069; *p* = 0.138). Importantly, the relatively wide confidence interval around the pooled estimate reflects imprecision and considerable uncertainty regarding the true magnitude of the effect. Although statistical heterogeneity was low (Q = 3.436; *p* = 0.179) and the I^2^ value remained at levels consistent with minimal variability, the limited number of studies means that estimates of heterogeneity may be unstable. Therefore, findings related to mindfulness interventions should be interpreted cautiously and considered preliminary until further well-designed randomized controlled trials provide more robust evidence (Figure 10).

**Figure 11:**
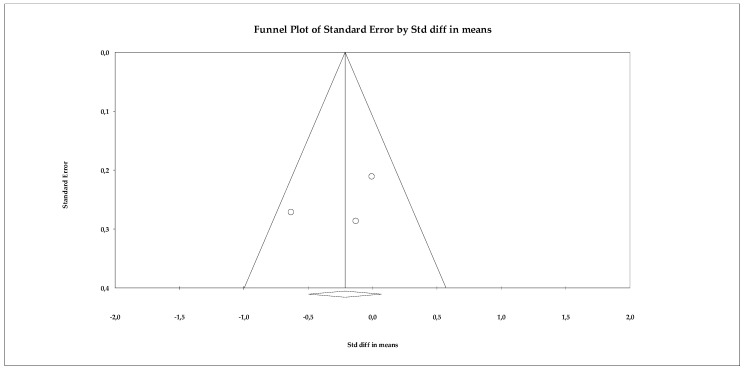
Funnel plot for the meta-analysis of mindfulness, used to assess publication bias.

The publication bias analysis showed no relevant evidence of distortion. The funnel plot (Figure 11) exhibited a relatively symmetrical distribution of studies, despite their limited number. The Begg–Mazumdar test yielded a tau of −0.333 (*p* = 0.601), while the Egger regression produced an intercept of −4.737 (two-tailed *p* = 0.586), suggesting the absence of significant publication bias. Additionally, the Trim and Fill method did not identify missing studies and kept the observed effect size unchanged (adjusted SMD = −0.213), which reinforces the stability of the results obtained.

##### Stress

**Figure 12:**
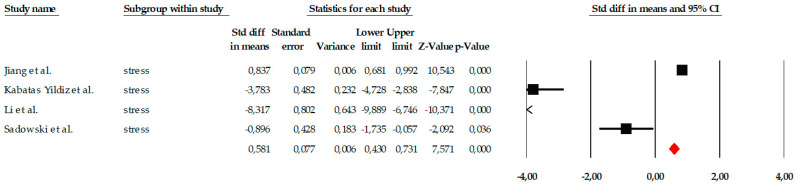
Forest plot of the meta-analysis on the effects of psychological interventions on perceived stress, based on the random-effects model. Black squares represent the standardized mean difference (SMD) for each individual study, with the size of the square proportional to the study weight. Horizontal lines indicate the 95% confidence intervals (CI). The red diamond represents the pooled effect estimate, and its width corresponds to the 95% CI. The vertical line at zero indicates no effect.

The analyses of the stress variable included four studies that evaluated the impact of different psychological interventions on reducing stress symptoms in older adults. The results of the fixed-effects model showed a large and statistically significant overall effect (SMD = 0.581; SE = 0.077; 95% CI = 0.430 to 0.731; *p* < 0.001), indicating that the interventions produced a substantial reduction in stress compared to the control groups. As shown in the forest plot (Figure 12), three studies reported very large and significant effects, while one showed a smaller but still significant effect, suggesting some variability in magnitude, although all studies agree in the direction of benefit. Marked heterogeneity was observed among the individual estimates, although the fixed-effects model was statistically significant in all cases. Standardized values ranged from SMD = −8.317 to SMD = 0.857, reflecting the existence of studies with particularly extreme effects. However, the consistency in the direction of the effect supports the robustness of the overall conclusion.

**Figure 13:**
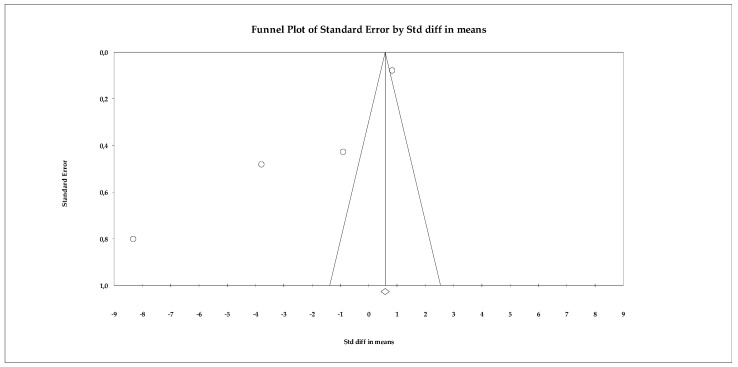
Funnel plot for the meta-analysis of perceived stress, used to assess publication bias.

Regarding publication bias, the funnel plot (Figure 13) showed some visual asymmetry, particularly due to one study with an extremely large effect size. Statistical tests provided additional information. Begg’s test reported a marginally significant correlation (tau = −1.000; *p* = 0.041, uncorrected), which could indicate asymmetry in effect sizes. The continuity-corrected test reduced this signal (*p* = 0.089), placing it just above the significance threshold. Egger’s test indicated a trend toward asymmetry (intercept = −9.985; *p* = 0.054), coming very close to the 0.05 significance level. Classic fail-safe N analysis determined that 21 additional studies with null effects would be needed to eliminate overall significance, suggesting that the results are relatively robust against archiving bias. Duval and Tweedie’s trim-and-fill method did not impute missing studies, maintaining the same overall estimator (SMD = 0.580), which supports the stability of the model.

##### Quality of Life

**Figure 14:**
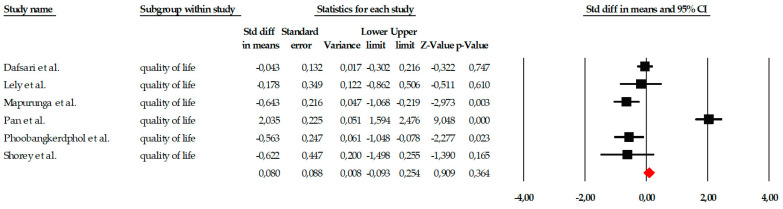
Forest plot of the global meta-analysis on the effects of psychological interventions on quality of life, estimated using a random-effects model. Black squares represent the standardized mean difference (SMD) for each individual study, with the size of the square proportional to the study weight. Horizontal lines indicate the 95% confidence intervals (CI). The red diamond represents the pooled effect estimate, and its width corresponds to the 95% CI. The vertical line at zero indicates no effect.

The quantitative analysis included six studies that assessed the effect of mindfulness-based interventions on quality of life. The fixed-effects model showed a very small and non-significant overall effect size (SMD = 0.080; SE = 0.088; 95% CI = −0.093 to 0.254; *p* = 0.364), indicating that, overall, the interventions did not produce detectable improvements in quality of life. Individual effect estimates showed heterogeneous effects: some studies showed moderate differences favoring the intervention (e.g., Mapurunga et al., SMD = −0.643; *p* = 0.003, or Phoobangkok et al., SMD = −0.539; *p* = 0.023), while others indicated no or even reversed effects. The results are summarized in the forest plot (Figure 14), where it can be observed that the confidence intervals of most studies cross the no-effect line.

**Figure 15:**
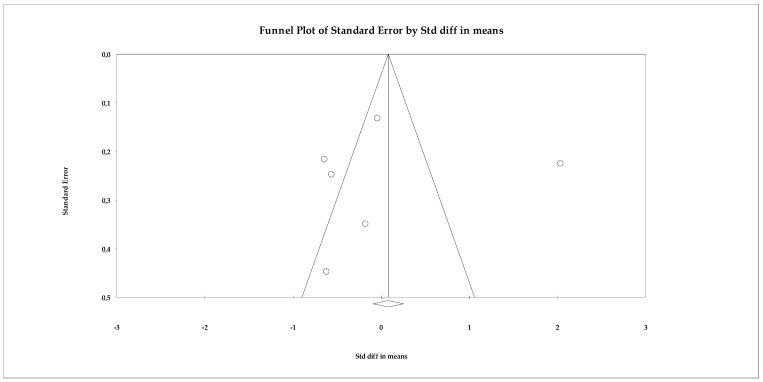
Funnel plot for the meta-analysis of quality of life, used to assess publication bias.

The publication bias analysis showed no evidence of asymmetry. The funnel plot (Figure 15) showed a relatively symmetrical distribution of studies around the overall effect size. This pattern was confirmed by the statistical analyses: Begg’s test was not significant (tau = 0.066; *p* = 0.85), indicating no correlation between study size and effect size. Similarly, Egger’s test also showed no evidence of bias (intercept = −0.615; *p* = 0.916). The classic fail-safe N statistic did not suggest a “file drawer” risk, as the number of studies hypothetically needed to cancel the effect did not exceed the total available, while the trim-and-fill method did not impute missing studies, reaffirming the stability of the model.

## 4. Discussion

The aim of this systematic review and meta-analysis was to synthesize the available evidence on the effectiveness of psychological interventions for improving mental health and quality of life in older adults. Overall, the findings indicate that psychological interventions are associated with consistent and robust improvements in depressive symptoms and perceived stress, while the effects on anxiety and quality of life are more variable and less consistent across studies. Although psychological interventions have been widely documented as beneficial in older populations, the present review contributes to the literature by providing an updated synthesis of recent randomized controlled trials that simultaneously examines multiple interrelated mental health outcomes. The quantitative analyses revealed small to moderate favorable effects overall, supporting the use of psychological interventions as safe and accessible approaches for promoting emotional well-being in later life. However, the heterogeneity of findings, particularly for anxiety and quality of life, highlights the importance of cautious interpretation and underscores the need for further well-powered studies to clarify the conditions under which broader benefits may be achieved.

Anxiety is one of the most frequent emotional problems in older adults and is often associated with chronic illnesses, functional decline, and loneliness (Araújo et al., 2025). In the present meta-analysis, although the overall direction of effects favored psychological interventions, the subgroup analysis for anxiety, which included a limited number of studies, did not reach statistical significance under the random-effects model. This finding suggests that evidence for anxiety reduction in later life remains less robust and more heterogeneous than for other outcomes such as depression or stress. Nevertheless, previous systematic reviews and meta-analyses have consistently reported beneficial effects of psychological interventions on anxiety symptoms in older adults. For example, S. Y. H. Li and Bressington (2019) found that mindfulness-based stress reduction (MBSR) was associated with significant reductions in anxiety, depression, and stress. Similarly, Wuthrich et al. (2021) reported significant effects of psychological treatments for co-occurring affective and anxiety disorders in older adults, and Wang et al. (2024) observed reductions in anxiety symptoms (SMD ≈ −0.33) in older adults with subclinical depression. Evidence from the general adult population also supports moderate effects of mindfulness-based interventions on anxiety (Hofmann et al., 2010). Differences between the present findings and previous reviews may partly reflect methodological and clinical variability across studies. The current review focused exclusively on randomized controlled trials published within the last five years, which reflects recent developments in intervention design, implementation fidelity, and outcome measurement. Additionally, several included studies evaluated relatively intensive or multimodal psychological interventions and involved participants with elevated baseline symptom levels, conditions that may influence observed effect sizes. However, the small number of studies included in the anxiety subgroup analysis, combined with the extremely high heterogeneity, likely contributed to unstable pooled estimates. Furthermore, variations in outcome definitions, scale orientation, and analytic decisions across reviews may also account for differences in reported effect magnitudes.

Regarding depression, the results of our meta-analysis indicate that psychological interventions are associated with significant reductions in depressive symptoms, although accompanied by substantial heterogeneity across studies. This finding is consistent with previous meta-analyses reporting moderate to large effects of psychological treatments for depression in older adults (S. Y. H. Li & Bressington, 2019; Wuthrich et al., 2021; Wang et al., 2024). Importantly, the high heterogeneity observed (I^2^ > 90%) suggests that the magnitude of benefit varies considerably depending on intervention type, delivery format, and study context. Descriptively, larger and more consistent effects were observed in interventions explicitly targeting depressive symptoms, particularly cognitive behavioral therapies, behavioral activation, and structured reminiscence-based programs. Interventions delivered in group formats and those involving participants with elevated baseline depressive symptoms also tended to report stronger effects, in line with previous findings (Wuthrich et al., 2021; Wang et al., 2024). In contrast, studies evaluating broader or multimodal psychosocial interventions, lower-intensity formats, or heterogeneous clinical populations often reported smaller or more variable effects. Differences in intervention duration, therapist involvement, outcome measures, and follow-up periods further contributed to between-study variability. Taken together, these findings suggest that while psychological interventions are generally effective for reducing depression in older adults, their impact is context-dependent. The observed heterogeneity highlights the importance of matching intervention type and intensity to clinical needs and underscores the value of targeted, disorder-specific approaches when treating late-life depression.

Perceived stress, understood as the subjective appraisal of life’s demands and the capacity to cope with them (Hofmann et al., 2010), also showed reductions following psychological interventions, particularly those based on relaxation, breathing, mindfulness, and coping skills training. In the present meta-analysis, these improvements were reflected by positive standardized mean differences, consistent with the directionality of the stress scales used, where higher scores indicate greater stress and positive effect sizes therefore represent stress reduction favoring the intervention group. This finding is supported by previous empirical evidence. For example, the systematic review and meta-analysis by Verhaeghen et al. (2025) (Cuijpers et al., 2006) concluded that mindfulness-based interventions are consistently associated with improvements in mental health and well-being in older adults. Similarly, a more recent meta-analysis focusing specifically on perceived stress in later life reported overall benefits of several intervention types, including reminiscence therapy and physical exercise (Morgado et al., 2024). From a theoretical perspective, mechanisms derived from coping and emotion regulation frameworks provide a coherent explanation for these effects. Mindfulness-based practices promote cognitive reappraisal, reduce rumination, modulate physiological arousal, and foster a greater sense of perceived control, processes that have been shown to mediate reductions in stress levels (Wuthrich et al., 2024). Taken together, these findings support the interpretation that psychological interventions constitute valid, useful, and empirically supported options for reducing perceived stress in older adults, despite differences in effect size directionality across outcomes due to scale orientation.

Finally, quality of life, a key dimension when evaluating the effectiveness of interventions in older adults (Huh et al., 2021), showed a more heterogeneous pattern of results. Although several individual studies reported improvements in specific quality-of-life domains, the pooled meta-analytic estimate did not reach statistical significance, indicating that these effects were not consistent across studies. This discrepancy likely reflects substantial variability in intervention types, outcome measures, study populations, and follow-up periods. Previous meta-analyses have reported significant improvements in quality of life following psychological interventions in older adults, particularly in the context of life review or well-being–focused interventions (Verhaeghen et al., 2025). Additional reviews have also described positive effects in specific settings, such as long-term care facilities or among older adults with chronic conditions (Hervás & Cebolla Ausiàs, 2016; van Leeuwen et al., 2019). However, the findings of the present meta-analysis suggest that, when these diverse interventions and measures are pooled, the overall effect on quality of life is not robust. Taken together, these results indicate that psychological interventions may improve certain dimensions of quality of life in specific contexts, but the current evidence does not support a stable and generalizable pooled effect. This highlights the need for future studies using standardized quality-of-life measures and longer follow-up periods to clarify the conditions under which meaningful improvements can be achieved.

Taken together, the findings of this review highlight the importance of adopting a multidimensional approach to mental health in older adults. Anxiety, depression, perceived stress, and quality of life represent distinct but overlapping dimensions that interact dynamically across the aging process. Psychological interventions appear to exert their effects not only by alleviating specific symptoms, but also by strengthening emotional regulation, coping capacity, and subjective well-being, which may in turn contribute to broader improvements in daily functioning and quality of life. This integrative perspective helps explain why interventions targeting emotional and cognitive processes can produce benefits across multiple outcome domains, even when these are measured separately.

This systematic review and meta-analysis has several limitations that should be considered when interpreting the results. First, considerable heterogeneity was observed among the included studies, both in the type of psychological intervention (e.g., mindfulness, reminiscence, cognitive behavioral therapy, psychoeducation, or relaxation) and in its duration, intensity, and format (individual, group, or online). This methodological variability may have influenced the magnitude of the observed effects and makes direct comparison between specific interventions difficult. Although appropriate statistical models were used to handle this heterogeneity, differences in study designs, contexts, and participant profiles could limit the generalizability of the results. Second, the literature search did not include certain specialized databases highly relevant to psychological intervention research, such as PsycINFO and the Cochrane Library, nor did it systematically search the grey literature. Although the databases used (PubMed, Scopus, CINAHL, and Web of Science) provide broad and multidisciplinary coverage of health and behavioral sciences, it is possible that some relevant unpublished or non-indexed studies were not captured, which may contribute to publication bias. In addition, the search strategy was restricted to studies published in English, potentially excluding relevant evidence reported in other languages. Third, several included studies had small sample sizes, which may compromise the precision of effect estimates and increase vulnerability to Type I and Type II errors. Furthermore, some trials did not clearly report key methodological aspects such as allocation concealment, blinding of outcome assessors, or handling of missing data. Consistent with this, the RoB-2 assessment indicated that a substantial proportion of studies presented some concerns or a high risk of bias, primarily due to limitations in reporting and methodological rigor. Fourth, although efforts were made to avoid duplication, there is a possibility of partial sample overlap across publications derived from the same trials, which could not always be definitively ruled out based on the information reported in the original articles. Another limitation of this review is the absence of a formal assessment of the certainty of evidence using the GRADE approach. Although methodological quality and risk of bias were evaluated using PEDro and RoB-2 tools, the lack of GRADE assessment limits the ability to provide a comprehensive evaluation of the overall strength and certainty of the evidence across outcomes. Future systematic reviews should incorporate GRADE methodology to enhance interpretability and support evidence-based decision-making. Finally, most studies assessed only short-term effects immediately after the intervention ended, resulting in uncertainty regarding the durability of the observed benefits for anxiety, depression, perceived stress, and quality of life. The lack of long-term follow-up represents a particularly important limitation in interventions targeting older adults, where sustained effects are critical for clinical and public health relevance.

## 5. Conclusions

This systematic review and meta-analysis demonstrates that psychological interventions exert a significant beneficial impact on the emotional health of older adults, with the most consistent and clinically meaningful improvements observed in depression and perceived stress. The reduction in depressive symptoms remained robust even after adjustment for publication bias, and stress showed a large and statistically significant effect across studies. In contrast, the evidence for anxiety, loneliness, mindfulness attention awareness, and quality of life was less consistent. For several of these outcomes, pooled effects under random-effects models were small or not statistically significant, and results varied substantially depending on the type of intervention, study design, and population characteristics. These findings indicate that, while some individual studies reported improvements in these domains, the overall evidence does not support a uniform or reliable effect across trials. Although the overall pooled effect across emotional variables was moderate and statistically significant, the very high heterogeneity highlights the marked methodological and clinical diversity among studies. This suggests that psychological interventions can be beneficial in older adults, but their effectiveness is highly context-dependent and sensitive to intervention characteristics. Importantly, publication bias analyses did not reveal systematic distortions, and trim-and-fill corrections only minimally affected effect sizes, supporting the robustness of the primary findings while underscoring the need for cautious interpretation of secondary outcomes.

## Figures and Tables

**Table 1:** Methodological quality of the included articles.

	1	2	3	4	5	6	7	8	9	10	11	Total Score
Aisenberg-Shafran and Shturm (2022)	1	1	0	1	0	0	1	1	0	1	1	6
Alexopoulos et al. (2021)	1	1	1	1	0	0	1	1	1	1	1	8
Dafsari et al. (2023)	1	1	1	0	0	1	1	1	1	1	1	8
Dutcher et al. (2025)	1	1	1	1	0	0	1	1	1	1	1	8
Hanaoka et al. (2025)	1	1	1	0	0	0	1	0	0	1	1	5
Javadzade et al. (2024)	1	1	0	0	0	0	0	1	0	1	1	4
Javadzade et al. (2025)	1	1	0	0	0	0	0	0	0	1	1	4
Jiang et al. (2025)	1	1	1	1	0	0	1	0	1	1	1	7
Kabataş Yıldız and Orak (2023)	1	1	0	1	0	0	0	1	0	1	1	5
Komase et al. (2025)	1	1	0	1	0	0	1	0	0	1	1	6
Kwok et al. (2024)	1	1	1	1	0	0	1	1	1	1	1	8
Landreville et al. (2025)	1	1	0	1	0	0	0	0	1	1	1	5
Lee et al. (2022)	1	1	0	1	0	0	1	1	1	1	1	7
Lely et al. (2022)	1	1	1	1	0	0	0	1	1	1	1	7
S. Li et al. (2022)	1	1	1	1	0	0	1	1	1	1	1	8
Lindsay et al. (2022)	1	1	1	1	0	0	1	0	1	1	1	7
Lwi et al. (2023)	1	1	0	1	0	0	0	1	0	1	1	5
Mapurunga et al. (2025)	1	1	0	1	0	0	0	0	0	1	1	4
Nordgren et al. (2024)	1	1	0	1	0	0	1	0	1	1	1	6
Pan et al. (2024)	1	1	1	1	0	0	1	0	1	1	1	7
Phoobangkerdphol et al. (2022)	1	1	1	1	0	0	1	1	1	1	1	8
Rodríguez-Romero et al. (2021)	1	1	0	0	0	0	0	0	0	1	1	3
Sadowski et al. (2025)	1	1	0	0	0	0	0	1	0	1	1	4
Shapira et al. (2021)	1	1	0	1	0	0	0	1	0	1	1	5
Shih et al. (2021)	1	1	0	1	0	0	0	1	0	1	1	5
Shorey et al. (2021)	1	1	0	1	0	0	0	0	0	1	1	4
Van Orden et al. (2021)	1	1	0	1	0	0	0	0	0	1	1	4
Wu et al. (2025)	1	1	0	1	0	0	1	1	1	1	1	7

Items: 1: eligibility criteria; 2: random allocation; 3: concealed allocation; 4: baseline comparability; 5: blind subjects; 6: blind therapists; 7: blind assessors; 8: adequate follow-up; 9: intention-to-treat analysis; 10: between-group comparisons; 11: point estimates and variability; yes = 1; no = 0.

**Table 2:** RoB-2 to assess the risk of bias.

BIAS	1	2	3	4	5	6
Aisenberg-Shafran and Shturm (2022)	High risk	High risk	Low or unclear risk	Low risk	Low or unclear risk	Unclear risk
Alexopoulos et al. (2021)	Low risk	Unclear risk	Low risk	Low risk	Unclear risk	Low or unclear risk
Dafsari et al. (2023)	Low risk	Unclear risk	Low risk	Low risk	Low risk	Low risk
Dutcher et al. (2025)	Low risk	Low risk	Low risk	Low risk	Low risk	Low risk
Hanaoka et al. (2025)	Low risk	Unclear risk	High risk	Low risk	Low risk	Unclear risk
Javadzade et al. (2024)	Unclear risk	High risk	Unclear risk	Unclear risk	Low risk	Unclear risk
Javadzade et al. (2025)	Unclear risk	High risk	Unclear risk	Unclear risk	Low risk	Unclear risk
Jiang et al. (2025)	Low risk	Unclear risk	Unclear risk	Low risk	Low risk	Low or unclear risk
Kabataş Yıldız and Orak (2023)	Unclear risk	High risk	Unclear risk	Unclear risk	Low risk	Unclear risk
Komase et al. (2025)	Unclear risk	Low risk	High risk	Low risk	Low risk	Unclear risk
Kwok et al. (2024)	Low risk	Low risk	Low risk	Low risk	Low risk	Low risk
Landreville et al. (2025)	Low risk	High risk	Low risk	Unclear risk	Low risk	Unclear risk
Lee et al. (2022)	Unclear risk	High risk	Low risk	Low risk	Low risk	Unclear risk
Lely et al. (2022)	Low risk	Low risk	Low risk	Unclear risk	Low risk	Low or unclear risk
S. Li et al. (2022)	Low risk	Unclear risk	Low risk	Low risk	Low risk	Low or unclear risk
Lindsay et al. (2022)	Low risk	Low risk	Unclear risk	Low risk	Low risk	Low or unclear risk
Lwi et al. (2023)	Unclear risk	High risk	Low risk	Unclear risk	Low risk	Unclear risk
Mapurunga et al. (2025)	Unclear risk	High risk	Unclear risk	Unclear risk	Los risk	Unclear risk
Nordgren et al. (2024)	Low risk	Low risk	Low risk	Low risk	Low risk	Low risk
Pan et al. (2024)	Low risk	Unclear risk	Low risk	Low risk	Low risk	Low or unclear risk
Phoobangkerdphol et al. (2022)	Low risk	Low risk	Low risk	Low risk	Low risk	Low risk
Rodríguez-Romero et al. (2021)	Unclear risk	High risk	Unclear risk	High risk	Low risk	Unclear or high risk
Sadowski et al. (2025)	Unclear risk	High risk	Unclear risk	Unclear risk	Unclear risk	Unclear or high risk
Shapira et al. (2021)	Unclear risk	High risk	Low risk	Unclear risk	Low risk	Unclear risk
Shih et al. (2021)	Unclear risk	Low risk	Unclear risk	Unclear risk	Low risk	Unclear or low risk
Shorey et al. (2021)	Unclear risk	High risk	Unclear risk	Unclear risk	Low risk	Unclear risk
Van Orden et al. (2021)	Unclear risk	High risk	Unclear risk	Unclear risk	Low risk	Unclear risk
Wu et al. (2025)	Unclear	High risk	Low risk	Low risk	Low risk	Unclear risk

Items: 1. Bias in randomization; 2. Bias due to deviations from the intervention; 3. Bias due to missing data; 4. Bias in the measurement of outcomes; 5. Bias in the selection of reports; 6. Overall assessment of the risk bias.

**Table 3:** Characteristics of the included studies.

Author	Sex	Sample CG/IG	Control Group	Age	Intervention Group		
					Treatment	Exercise Parameters	Results
Aisenberg-Shafran and Shturm (2022)	F: 92% M: 8%	8/16	Care as usual	73.13	Mindfulness-Based Intervention for Seniors (MBIS) and Cognitive Behavioral Therapy (CBT)	F: 1 times/week #S: 8 sessions D: 30 min	Both interventions improved attitudes toward psychological treatment versus control; MBIS reduced worry and CBT reduced anxiety.
Alexopoulos et al. (2021)	F: 20% M: 80%	120/129	Problem-Solving Therapy (PST)	70.2	Engage psychotherapy	F: 1 times/week #S: 9 sessions D: 90 min	Both treatments reduced Hamilton Depression Rating Scale (HAM-D) scores over 9 weeks, with no significant differences between Engage psychotherapy and PST; response and remission rates were similar, supporting the non-inferiority of Engage.
Dafsari et al. (2023)	F: 66% M: 34%	114/115	Supportive Unspecific Intervention (SUI)	70.2	Late-Life Depression Cognitive Behavioral Therapy (LLD-CBT)	F: 2 times/week #S: 15 sessions D: NA	No significant between-group differences were observed in Geriatric Depression Scale (GDS) scores at post-treatment; both LLD-CBT and supportive unspecific treatment showed improvements over time, with no evidence of superiority of LLD-CBT.
Dutcher et al. (2025)	F: 79% M: 21%	97/93	Health Enhancement Program (HEP)	69.78	Mindfulness-Based Stress Reduction (MBSR)	F: 1 times/week #S: 8 sessions D: 120 min	MBSR and HEP both reduced loneliness (main effect of time: with no significant difference between conditions. These findings suggest that both MBSR and HEP may be promising approaches to addressing loneliness in older adults with effects lasting for months.
Hanaoka et al. (2025)	F: 93% M: 7%	31/30	Reminiscence therapy using themed conversations	78.99	Reminiscence therapy using smells	F: 1 times/week #S: 8 sessions D: 40 min	Both MBSR and HEP were associated with significant reductions in loneliness over time; however, no significant differences were observed between the two interventions, and effects were maintained for several months. After adjusting for baseline differences in olfactory Visual Analogue Scale (VAS) scores, the intervention showed significant effects on loneliness (UCLA scores). Improvements were associated with participants’ perceived usefulness of olfactory cues for inducing reminiscence.
Javadzade et al. (2024)	F: 60% M: 40%	30/30	Control group	66.5	MBSR	F: 1 times/week #S: 8 sessions D: 90 min	Compared with the control group, MBSR significantly reduced depressive symptoms and improved emotion regulation and sleep quality in older adults with depression.
Javadzade et al. (2025)	F: M:	30/30	Control group	66.5	MBSR	F: 1 times/week #S: 8 sessions D: 90 min	Depressive symptoms significantly decreased after 8 weeks of MBSR, whereas no significant improvements were observed in physical symptoms Powell and Enright Physical Symptoms Inventory (PE-PSI).
Jiang et al. (2025)	F: 73% M: 27%	356/795	Telephone befriending/support calls	76.46	Telephone-delivered behavioral activation Telephone-Delivered Mindful-ness	F: 2 times/week #S: 8 sessions D: 30 min	Compared with Telephone Befriending/support calls (Tele-BF), Telephone-delivered Behavioral Activation (Tele-BA) significantly reduced loneliness (UCLA-LS) and showed additional benefits in perceived social support, stress, psychological well-being, depression, and anxiety, whereas Telephone-delivered Mindfulness (Tele-MF) showed more limited and less consistent effects across outcomes.
Kabataş Yıldız and Orak (2023)	F: 27% M: 73%	24/24	Control group	76.04	MBSR	F: 1 times/week #S: 8 sessions D: 45 min	The MBSR program led to substantial reductions in perceived stress (≈55% overall, ≈50% across sub-dimensions) and a moderate reduction in geriatric depression scores (≈14%). A significant between-group difference was observed in Positive Reminiscence Scale scores, with a small-to-moderate advantage for reminiscence therapy based on memories of enjoyment compared with memories of hard work.
Komase et al. (2025)	F: 83% M: 17%	14/12	Reminiscence therapy based on memories of enjoyment	80.73	Reminiscence therapy based on hard work	F: 1 times/week #S: 4 sessions D: 30–50 min	A significant between-group difference was observed in Positive Reminiscence Scale scores, with a small-to-moderate effect favoring reminiscence therapy based on memories of enjoyment over memories of hard work.
Kwok et al. (2024)	F: 73% M: 27%	356/795	Telephone befriending/support calls	76.46	Telephone-delivered behavioral activation Telephone-Delivered Mindful-ness	F: 2 times/week #S: 8 sessions D: 30 min	Compared with befriending, both behavioral activation and mindfulness reduced loneliness on the UCLA Loneliness Scale at three months, while reductions on the De Jong Gierveld scale were observed only in the mindfulness group. Both interventions improved sleep quality, whereas perceived stress increased. Behavioral activation additionally improved psychological well-being and perceived social support, with no significant between-group differences in depression, life satisfaction, or social network.
Landreville et al. (2025)	F: 89% M: 11%	75/75	Wait-list control group	68.18	Cognitive Behavioral Therapy—Self-Help (CBT-SH)	F: 1 times/week #S: 15 sessions D: 30 min	Compared with the wait-list control, the intervention produced greater improvements in primary and secondary outcomes with small-to-large effect sizes, which were maintained or further improved at follow-up; participants in the control group also showed improvements after receiving the intervention.
Lee et al. (2022)	F: 84% M: 16%	104/105	Wait-list control group	71.45	modified Mindfulness-Based Stress Reduction (mMBSR)	F: 1 times/week #S: 8 sessions D: 120 min	Baseline characteristics were comparable between groups except for sleep quality. At two months, the intervention group showed improvements in mental well-being, depressive symptoms, cognitive function, and sleep quality; all effects except mental well-being were sustained at four months.
Lely et al. (2022)	F: 25% M: 75%	16/17	Present-Centered Therapy (PCT)	63.81	Narrative Exposure Therapy (NET)	F: 1–2 times/week #S: 11 sessions D: NA minutes	No significant between-group effects were observed at post-treatment or follow-up; however, medium-to-large within-group improvements in psychopathology were found in the NET group at follow-up, with no comparable changes in the PCT group. Resilience-related outcomes did not change significantly in either group.
S. Li et al. (2022)	F: 63% M: 37%	30/30	Control group	65.7	Chinese Traditional Festival Activities—Group Reminiscence Therapy (CTFA-GRT)	F: 1 times/week #S: 32 sessions D: 60–240 min	CTFA-GRT reduced perceived stress and loneliness in rural older adults living alone, with effects emerging several months after baseline and sustained following the intervention.
Lindsay et al. (2022)	F: 78% M: 22%	97/93	HEP	69.77	MBSR	F: 1 times/week #S: 8 sessions D: 120 min	Mindfulness training was associated with greater increases in stimulated Interleikin-6 (IL-6) production over time compared with the control program, from pre- to post-intervention and to follow-up; no study-related adverse events were reported.
Lwi et al. (2023)	F: 55% M: 45%	26/23	Brain Health education	72.8	MBSR	F: 1 times/week #S: 8 sessions D: 150 min	Both interventions showed high recruitment, satisfaction, and retention rates, with similar levels of preliminary efficacy.
Mapurunga et al. (2025)	F: 84% M: 16%	46/50	Active control group (computer-based cognitive stimulation)	74.6	Mindfulness-Based Health Promotion; (MBHP)	F: 1 times/week #S: 8 sessions D: 150 min	Quantitative improvements in Quality of Life (QOL) were observed only in the active control group, whereas the MBHP group showed improvements in stress, anxiety, intrinsic religiosity, and sleep quality. Qualitative findings suggested perceived gains in social support, self-awareness, self-care, and sleep quality in the MBHP group, indicating a discrepancy between quantitative QOL outcomes and qualitative reports.
Nordgren et al. (2024)	F: 71% M: 29%	51/50	Active control group	71.9	Internet-delivered Cognitive Behavioral Therapy (ICBT)	F: 1 times/week #S: 10 sessions D: 15 min	Both groups showed significant reductions in depression, with the treatment group demonstrating greater improvements on the GDS-15 and Beck Depression Inventory (BDI-II) (moderate-to-large and moderate effects, respectively), but not on the Patient Health Questionnaire (PHQ-9).
Pan et al. (2024)	F: 62% M: 38%	60/60	Control group	85.5	Resourcefulness-Based Instrumental Reminiscence Therapy (RBIRT)	F: 1 times/week #S: 6 sessions D: 60 min	Compared with the control group, the intervention significantly improved psychological adjustment, resourcefulness, self-efficacy, social support, and mental health–related quality of life, with effects maintained at follow-up.
Phoobangkerdphol et al. (2022)	F: 85% M: 15%	33/35	Balance training	68.9	Walking meditation	F: 5–7 times/week #S: 120 sessions D: 20–30 min	No significant between-group differences were observed in functional performance (Timed Up and Go Test, Functional Research Test, Single Leg Stand Test) or quality-of-life and mental health measures (EuroQOL 5 dimensions 5 levels and Thai Geriatric Mental Health Assessment Tool-15). Adherence to the exercise protocol was high, and no serious adverse events were reported.
Rodríguez-Romero et al. (2021)	F: 87% M: 13%	26/29	Control group	80.6	educational workshops, mindfulness, yoga, walking and visits to urban gardens	F: 1–2 times/week #S: 18 sessions D: NA minutes	At six months post-intervention, the intervention group showed lower loneliness and greater improvements in social support (DUKE-UNC-11), mental health (Short Form-12), and depressive symptoms compared with the control group.
Sadowski et al. (2025)	F: 83% M: 17%	12/12	Psychoeducational pamphlet control group	75	Embodied and Embedded Mindfulness Compassion Framework—Virtual Reality (EEMCF-VR)	F: 2 times/week #S: 8 sessions D: 20 min	EEMCF-VR demonstrated good feasibility and acceptability, with low attrition and minimal adverse effects. Compared with controls, the intervention group showed significantly lower stress and negative emotions at follow-up, supported by positive qualitative feedback.
Shapira et al. (2021)	F: 80% M: 20%	18/64	Wait-list control group	71.9	Web-based platform cognitive behavioral and mindfulness	F: 2 times/week #S: 8 sessions D: 20 min	The intervention group showed clinically and statistically significant reductions in depression, maintained at one-month follow-up, and short-term reductions in loneliness that were not sustained. Social support showed non-significant increases, and no overall changes were observed in the wait-list control group.
Shih et al. (2021)	F: 88% M: 12%	29/28	Active control group	70.28	Mindfulness-based Cognitive Therapy (MCBT)	F: 1 times/week #S: 8 sessions D: 120 min	Both groups showed significant reductions in HAMD scores; however, only the MBCT group demonstrated substantial improvements in cognitive and mindfulness-related outcomes (AMT, RRS, MAAS).
Shorey et al. (2021)	F: 68% M: 32%	11/10	Control group	69.4	Where-there-is-no-psychiatrist Integrated Personal Therapy (WIPT)—integrated Solution-Focused Brief Therapy (SFBT)	F: NA #S: 7 sessions D: NA	No significant between-group differences were observed in depression, anxiety, life satisfaction, friendship, or quality of life; however, the intervention group showed improvements in quality-of-life scores from baseline to 6 months. At follow-up, the control group exhibited higher cortisol and lower annexin-A1 levels, whereas no such changes were observed in the intervention group.
Van Orden et al. (2021)	F: 68% M: 32%	30/32	Care as usual	72	Social Engage	F: 1 times/week #S: 10 sessions D: NA	Participants showed high compliance with the intervention. Social Engage did not improve belonging or perceived burden but was effective in reducing depressive symptoms and improving social–emotional quality of life.
Wu et al. (2025)	F: 74% M: 26%	26/52	Usual care	85.5	Group Reminiscence Therapy assisted by Memory Specificity Training (GRT-mest)	F: 1 times/week #S: 4 sessions D: 90 min	Compared with the other groups, GRT-mest produced greater improvements in depressive symptoms, autobiographical memory, and rumination, with effects maintained for several months; improvements in autobiographical memory showed only an indirect short-term association with reductions in depression.

## Data Availability

No new data were created or analyzed in this study.

## Supplementary Materials

The following supporting information can be downloaded at: https://www.mdpi.com/article/10.3390/ejihpe16030034/s1, Table S1: PRISMA 2020 Checklist.

## Abbreviations

The following abbreviations are used in this manuscript:

EG	Experimental Group
CG	Control Group
PSS	Perceived Stress Scale
SF-36	Short Form-36 Health Survey
PCS	Physical Component Summary
MCS	Mental Component Summary
GDS	Geriatric Depression Scale
SD	Standard Deviation
RCT	Randomized Controlled Trial
